# Artificial Intelligence-Driven Construction of Predictive and Druggable Frameworks to an In Silico Bioengineering Evidence Support for Therapy of Esophageal Squamous Cell Carcinoma Patients: Insights from a Toll-like Receptor Signal with Th17 and T Helper Microenvironment

**DOI:** 10.3390/bioengineering13060622

**Published:** 2026-05-26

**Authors:** Bo Liu, Jiazhou Xiao, Xuan Tao, Xu Li

**Affiliations:** 1 Department of Thoracic Surgery, The First Affiliated Hospital, Fujian Medical University, Fuzhou 350004, China; 2 Department of Thoracic Surgery, National Regional Medical Center, Binhai Campus of the First Affiliated Hospital, Fujian Medical University, Fuzhou 350212, China; 3 Department of Pathology, The First Affiliated Hospital, Fujian Medical University, Fuzhou 350212, China; 4 Department of Pathology, National Regional Medical Center, Binhai Campus of the First Affiliated Hospital, Fujian Medical University, Fuzhou 350212, China

**Keywords:** esophageal squamous cell carcinoma, Toll-like receptor, immune microenvironment, artificial intelligence, predictive model

## Abstract

Objective: Dysregulation of Toll-like receptor signaling and increased proportions of Th17 and other T helper cells can facilitate esophageal squamous cell carcinoma (ESCC) progression. Methods: By integrating WGCNA, Limma, and artificial intelligence (AI, including LASSO-Cox regression and SOM) frameworks, we first identified a Toll-like receptor signaling and Th17 and T helper cell (ThpT)-related prognostic model and Thp molecular subgroups for ESCC patients in bulk transcriptomic profiles. Next, Thp-associated hub genes were identified, followed by evaluation of corresponding molecular and immune features. Indeed, the heterogeneity of ESCC was estimated using a single-cell transcriptomic dataset acquired from the GEO database. Furthermore, we also evaluated Thp-associated hub gene molecular and biological functions in spatial and temporal manners on targeted cells via pseudotime trajectory and AI-driven targeted gene knockout (KO). ESCC therapeutic agents targeting Thp-associated hub genes were enriched via a drug–gene network and then examined by ridge regression-driven drug sensitivity estimation and molecular docking. To enhance the robustness of our study, we performed in vitro studies to quantify the relationship of the targeted gene with Th17 and ESCC progression. Results: Based on Thp, we successfully identified a prognostic model and molecular subgroups of ESCC patients. DDX39A and PBK should be considered ThpT-related hub genes involved in ESCC progression and decreased infiltration of Th17 cells. Based on drug sensitivity estimation and molecular docking, bleomycin and talazoparib may be potential drugs for treating esophageal squamous cell carcinoma. Conclusions: ThpT can guide personalized and precision medicine for ESCC patients. Our study provides a novel clinical translation strategy for combating ESCC.

## 1. Introduction

Esophageal Squamous Cell Carcinoma (ESCC) is one of the most lethal malignancies worldwide, particularly prevalent in Asia, with a staggering annual incidence of hundreds of thousands of cases in China alone [1]. The high mortality rate associated with ESCC underscores the urgent need for novel therapeutic strategies, as current treatment modalities, including surgery, radiotherapy, and chemotherapy, often yield suboptimal outcomes due to tumor heterogeneity and resistance [2]. Hence, there is an urgent need to identify key genes that can be used to predict prognosis and serve as novel therapeutic targets for ESCC.

Toll-like receptors (TLRs) are key members of pattern recognition receptors and play a central role in innate immune responses and the regulation of the tumor immune microenvironment [3]. TLRs, by recognizing pathogen-associated molecular patterns (PAMPs) and damage-associated molecular patterns (DAMPs), initiate downstream signal cascades dependent on MyD88 or TRIF, activate transcription factors such as NF-κB and IRF3, and thereby regulate the production of inflammatory cytokines and type I interferons [4]. In recent years, the dual role of TLR signaling in tumor occurrence and development has attracted much attention: on the one hand, TLR activation can promote the maturation of dendritic cells and antigen presentation, enhancing adaptive anti-tumor immune responses [5]; on the other hand, dysregulated TLR signaling can drive tumor malignancy progression by inducing chronic inflammation, promoting tumor cell proliferation and survival, and mediating immune escape [6]. In various malignant tumors, abnormal activation of TLR signaling is closely related to tumor progression and patient prognosis [7,8,9,10,11,12,13,14]. Studies have shown that continuous activation of TLR4 can promote tumor-associated macrophage infiltration in colon cancer, supporting tumor malignancy progression [15], while TLR6 is downregulated in colorectal cancer, and its activation can exert an anti-tumor effect by inhibiting NF-κB signaling [16]. In breast cancer, differences in TLR expression profiles are closely related to tumor immune infiltration characteristics and treatment responses [17]. These findings suggest that precise regulation of TLR signaling is crucial for maintaining immune homeostasis and inhibiting tumor progression. However, the relationship between the characteristics of the esophageal cancer immune microenvironment and TLR signaling regulation remains unclear.

Th17 cells are a unique subset of CD4 helper T cells, distinct from the classical Th1 and Th2 cell lineages. Harrington was the first to identify CD4 T cells secreting IL-17 in mice and subsequently named them Th17 cells [18]. The lineage-determining transcription factor RORγt and STAT3 jointly regulate their developmental program and functional stability [19]. The lineage orientation of Th17 cells is highly dependent on the cytokine environment: IL-6 and TGF-β synergistically promote Th17 polarization. Among them, the concentration of TGF-β is crucial—lower concentrations can promote RORγt expression and drive Th17 differentiation, while higher concentrations will inhibit RORγt and induce Foxp3, thereby promoting the development of regulatory T cells [20,21]. It is worth noting that TLR signaling regulates the differentiation and function of Th17 cells by influencing the cytokine profile of antigen-presenting cells. In the mouse model of cerebral hemorrhage, activation of TLR4 and TLR2 can induce the maturation of dendritic cells, reducing the proportion of Treg/Th17 cells through the p38-MAPK/MyD88 signaling pathway, exacerbating the neuroinflammatory response; while TLR9 exerts the opposite effect through the IDO1/GCN2 pathway, promoting Treg differentiation and inhibiting Th17 responses. In the human system, the optimal induction of Th17 cells requires stimulation of T cells by a TLR-activated monocyte environment, depending on cell-to-cell contact. Moreover, the activation of TLR4 and Dectin-2 synergistically promotes the induction of CD4+ T cells to differentiate into Th17 phenotypes. Th17 cells mainly exert pro-inflammatory functions by secreting their characteristic cytokine IL-17 [22]. The Th17/IL-17 axis has been confirmed to be associated with autoimmune diseases such as asthma, systemic lupus erythematosus, and rheumatoid arthritis and also plays an important regulatory role in tumor biology [23,24]. Th17 cells have strong anti-tumor potential, especially in adoptive cell therapy and vaccine strategies [25,26]. Tumor-specific Th17 cells, after adoptive transfer, can more effectively clear melanoma than other CD4+ T cell subsets and coordinate the host immune system to fight against distant metastasis through unique mechanisms [27]. Its anti-tumor mechanism includes collaborating with host B cells, inducing the formation of germinal centers and the production of tumor-reactive antibodies through the co-stimulation of IL-21 and CD40L, thereby driving a persistent tumor immune response [28]; in the ovarian cancer model, Th17-induced dendritic cell vaccines can remodel the tumor myeloid microenvironment, increase tumor Th17 cells, and make the tumor sensitive to PD-1 immune checkpoint blockade therapy [29]. Additionally, Stat5-deficient CD4+ naïve T cells tend to differentiate into IFN-γ+ and IFN-γ− Th17 cell subpopulations with stronger anti-melanoma activity, and their effector functions are achieved by enhancing Notch1 pathway activation [30]. These findings highlight the crucial role of Th17 cells as effector cells in stimulating a comprehensive anti-tumor immune response, although the mechanism by which Th17 cells infiltrate the immune microenvironment of esophageal cancer is not yet clear.

In our study, we first identified Toll-like receptor signaling and Th17 and T helper cell (ThpT)-associated DEGs via the Limma and WGCNA framework in the ESCC GEO bulk profile. Next, the deep learning algorithm (SOM) revealed the ThpT-associated molecular subgroup classification in the TCGA-ESCC bulk dataset. The results of Lasso-Cox regression indicated that a novel predictive model could be established for ESCC patients. Furthermore, multi-omic and pre-clinical studies proved that DDX39A and PBK are two hub genes that are involved in ESCC progression and increased proportions of Th17 and T helper cells. Moreover, bleomycin and talazoparib can be considered a therapeutic framework targeting DDX39A and PBK for the treatment of ESCC. Overall, our study revealed that ThpT can guide prediction of ESCC clinical outcomes and stratification of ESCC patients. We also found that DDX39A and PBK were closely linked to ESCC progression and immune escape of ESCC cells. In addition, ESCC patients may benefit from treatment with bleomycin and talazoparib.

## 2. Materials and Methods

**Figure 1:**
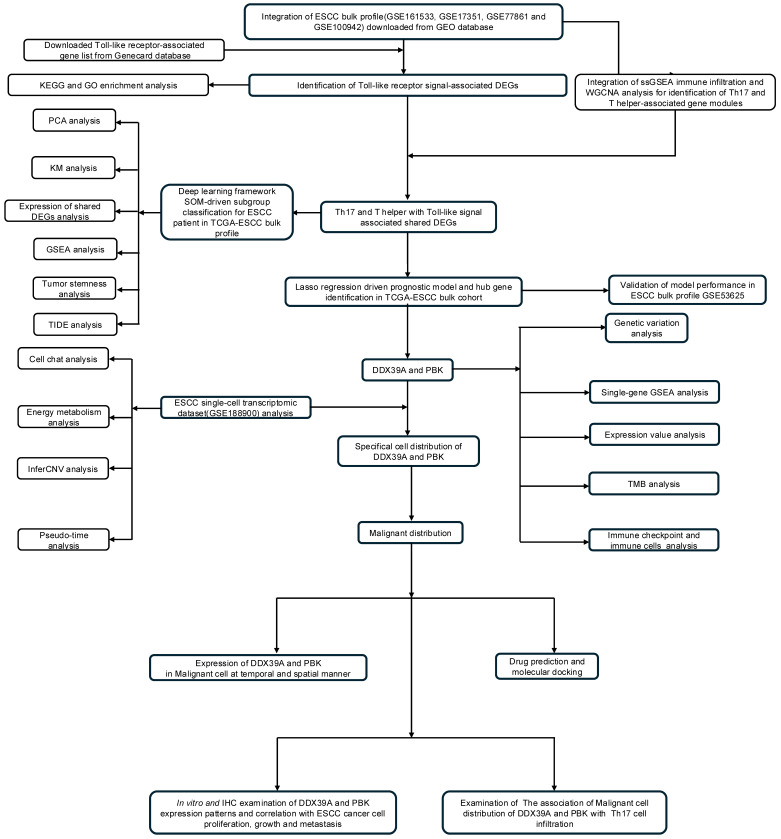
Flowchart of the study.

We described the workflow of this study in Figure 1.

### 2.1. Patient Samples

A total of 200 paraffin samples of esophageal squamous cell carcinoma were collected from the Department of Thoracic Surgery at the First Affiliated Hospital of Fujian Medical University (Fuzhou, China). All participants provided written informed consent during the recruitment process. Samples were stored at −80 °C or fixed in formalin. According to the revised International Esophageal Cancer Staging System, all cases were clinically and pathologically diagnosed with esophageal squamous cell carcinoma. These 200 formalin-fixed, paraffin-embedded (FFPE) samples from ESCC patients were employed for immunohistochemical (IHC) staining to achieve two main objectives: 1. To evaluate the protein expression levels of DDX39A and PBK in esophageal squamous cell carcinoma tissues; 2. To assess the correlation between DDX39A/PBK expression and the infiltration level of Th17 cells within the tumor microenvironment. This study was approved by the Ethics Committee of the First Affiliated Hospital of Fujian Medical University (Fujian Medical University Affiliated Hospital Ethics Medical Research (2024) No. 509).

### 2.2. Identification of DEGs

Four bulk cohorts of ESCC, namely GSE161533, GSE17351, GSE77861, and GSE100942, were sourced from the Gene Expression Omnibus (GEO) database via the GEOquery package of R [31]. To address missing values, the impute package of R was employed for data supplementation [32]. Additionally, to integrate bulk data and mitigate batch effects across the diverse datasets, the ComBat function from the sva and Limma packages of R was utilized [33,34]. During the annotation process, probes linked to multiple molecules were eliminated; in cases where probes corresponded to the same molecule, only the probe exhibiting the highest signal intensity was preserved. The threshold for significant DEGs in the integrated dataset was set with a threshold of |log2FC| > 0.5 and *p* < 0.05 via the Limma package of R [35]. The Toll-like receptor-associated gene list was acquired from the GeneCard database with a threshold of relevance score > 1. Finally, the Toll-like receptor-associated gene list was intersected with DEGs in the integrated dataset to obtain Toll-like receptor-associated DEGs. KEGG and GO enrichment analysis was conducted via the ClusterProfiler package of R in accordance with the hallmark gene set [36].

### 2.3. WGCNA

ssGSEA utilizes a deconvolution algorithm to estimate the composition and abundance of immune cell types within a cellular mixture based on transcriptomic data [37]. In this investigation, we initially evaluated the proportions of 22 distinct immune cell types in both normal and ESCC samples from the integrated bulk dataset. Weighted Gene Co-expression Network Analysis (WGCNA) was conducted to identify modules of highly correlated genes and elucidate the interconnections among these modules and their associations with external sample characteristics, ultimately aiming to pinpoint candidate biomarkers or therapeutic targets. In our study, WGCNA was executed using the R package WGCNA to identify modules that exhibited the strongest correlation with ESCC and T helper and Th17 cells in patients suffering from ESCC [38]. Initially, we preprocessed the sample data and eliminated any outliers. Following this, a correlation matrix was generated through the WGCNA software package [39]. The optimal soft threshold was determined to convert the correlation matrix into an adjacency matrix, from which a topological overlap matrix (TOM) was subsequently derived. The TOM-based phase dissimilarity metric was employed to group genes exhibiting similar expression patterns into gene modules via average linkage hierarchical clustering. The two modules demonstrated the highest correlation with T helper and Th17 cells. Ultimately, the Toll-like receptor-associated DEGs were intersected with the module shared by ESCC, T helper, and Th17 cells to identify ThpT-related shared DEGs.

### 2.4. Self-Organizing Maps (SOM) for Stratification

The self-organizing map (SOM) represents a type of artificial neural network algorithm that is applicable to character classification based on the Korhonen package of R [40]. We obtained STAR-counts data in conjunction with pertinent clinical information regarding ESCC tumors from the TCGA database. Following this, we converted the data into Transcripts Per Million (TPM) format and conducted normalization using the log2(TPM + 1) transformation, adhering to the previously described methodology [41]. Utilizing the TCGA-ESCC cohort as a basis, we employed SOM to identify novel molecular subgroups following established protocols [40]. Subsequent to the identification of diverse molecular subgroups within the TCGA-ESCC cohort, we conducted analyses of Kaplan–Meier (KM) plots, clinical significance, tumor stemness, tumor purity, and checkpoint expression across the identified subgroups.

### 2.5. Lasso-Cox Regression

To create predictive models associated with the ThpT-related shared DEGs to assess prognosis in ESCC and to pinpoint crucial variables, we employed the Lasso-Cox regression approach within the TCGA-ESCC cohort. This strategy was subsequently corroborated using the GSE53662 dataset. Additionally, in the process of constructing the prognostic model, we performed a comprehensive set of analyses, which included Kaplan–Meier (KM) survival analysis, risk factor evaluation, and time-dependent receiver operating characteristic (ROC) analysis in both the TCGA-ESCC and GSE53662 cohorts, utilizing the survival and rms packages available in R software (4.2.1).

### 2.6. Molecular and Immune Feature Estimation

Genetic variation analysis of targeted genes was performed using the CBioPortal database [42]. Single-gene GSEA of targeted genes was conducted with the ClusterProfiler package of R in accordance with the hallmark gene set [37]. Chromatin localization analysis of the targeted gene was assessed and visualized using the circlize package of R. The expression levels of targeted genes across clinical parameters were assessed using the UALCAN database [43]. The association of tumor mutation burden (TMB) with targeted genes was assessed and visualized using the ggstatsplot package of R. The relationship between immune cell proportions and targeted genes was assessed using the ssGSEA algorithm of R. Additionally, the association between immune checkpoint expressions and targeted genes was also evaluated using ggplot2 of R.

### 2.7. Single-Cell Transcriptomic Analysis

Initially, we acquired the single-cell transcriptomic dataset for ESCC, designated as GSE188900 (including 7 ESCC patients), from the GEO database. The analysis of single-cell RNA sequencing (scRNA-seq) data involved several steps, including quality control (QC), dimensionality reduction, and identification of marker genes, all of which were performed using the Seurat R package [44]. QC measures were applied to each individual cell, with established criteria that included gene counts ranging from 200 to 6000, a UMI count exceeding 1000, and a mitochondrial gene percentage of less than 10%. Following these QC processes, normalization of the data was conducted, enabling the identification of 2000 genes that exhibited significant variability for subsequent analysis. Post-normalization, dimensionality reduction techniques, specifically t-SNE and UMAP, were employed. Cell-type annotation was achieved through the implementation of the SingleR algorithm of R [45]. The expression levels of target genes were assessed within the various annotated cell populations. Intercellular communication networks were inferred using the Cell-phoneDB package of R [46]. Additionally, metabolic pathways at the single-cell level were investigated using the single-cell Metabolism package in R [47]. Single-cell Monocle2 pseudotime analysis was performed to decipher the role of the targeted gene at the single-cell level [48]. Moreover, upstream and downstream gene lists of targeted genes in targeted cells were obtained using scTenifoldKnk [49]. The GeneMANIA database was used for evaluation of upstream and downstream gene list interactions. In addition, KEGG and GO enrichment were performed to estimate molecular and biological functions among these genes.

### 2.8. Drug Prediction and Molecular Docking

A comprehensive approach combining network pharmacology and molecular docking techniques was employed to systematically explore potential interactions between drugs and their targets [50]. The prediction procedure was carried out using the R package pRRophetic [51]. The estimation of the samples’ half-maximal inhibitory concentration (IC50) was performed through ridge regression analysis [51]. Molecular docking was performed to assess interactions between drugs and proteins. The Protein Data Bank (PDB) files corresponding to the target proteins were sourced from the RCSB PDB. Ligand structures were retrieved in SDF format from the PubChem database. Subsequently, molecular docking was executed to estimate the binding affinities between the selected proteins and the compounds. Initially, the PyMOL software (Version 2.6.0) was utilized to eliminate water molecules and ligands, preserving only the protein backbone. Following this, the AutoDock Vina Tool (Version 4.2.6) was employed to identify potential binding sites on the protein surface and to conduct flexible molecular docking. This process involved calculating docking scores and binding affinities (Vina score in kcal/mol) for each identified binding site. The top five binding sites were ranked according to their binding energies, with the site exhibiting the lowest binding energy being selected for visualization in PyMOL. This visualization highlighted the positions of hydrogen bonds associated with ligand interactions in the resulting image. The results were subsequently illustrated in PyMOL to depict the binding modes and hydrogen bonding interactions.

### 2.9. Cell Lines and Culture

The cell lines NCI-H520, HET1, and HEK 293T were obtained from the Shanghai Academy of Biological Sciences in Shanghai, China. The NCI-H520 and HET1 lines were cultivated in Roswell Park Memorial Institute (RPMI) 1640 complete medium, which was augmented with a 1% antibiotic mixture consisting of penicillin and streptomycin, along with 10% fetal bovine serum (FBS, Gibco). Conversely, the HEK 293T cell line was maintained in Dulbecco’s Modified Eagle Medium (DMEM), similarly supplemented with a 1% penicillin-streptomycin mixture and 10% FBS. All procedures for cell passage were performed under strictly regulated conditions of 37 °C and 5% CO_2_ within a humidified incubator.

### 2.10. Western Blotting

The protein lysates extracted from cells and tissues (40 micrograms) were separated on an SDS-PAGE gel and transferred onto a PVDF membrane. The membrane was blocked, then incubated with the specified primary antibodies [anti-DDX39A (Abcam, Cambridge, UK, 1:1000), anti-PBK (Abcam, Cambridge, UK, 1:1000)] at 4 °C overnight, followed by incubation with the secondary antibody at room temperature for 2 h. The protein bands were visualized using the enhanced chemiluminescence (ECL) system and quantified using ImageJ software (version 1.54p; National Institutes of Health).

### 2.11. Silencing via shRNA

Lentiviral virions were produced by co-transfection of HEK293T cells with 8 μg PLKO1-puro vector and 5 μg packaging and envelope vectors using Lipofectamine 2000 (Invitrogen, Carlsbad, CA, USA) following the manufacturer’s protocol. The lentivirus virions were then harvested 48 h after transfection. KYSE150 cells were separately infected with lentivirus containing knockdown and CON for 24 h. Two days later, the virus-infected cells were then selected using 4 μg/mL puromycin (Sigma-Aldrich; Merck KGaA, Darmstadt, Germany) for 48 h and subjected to required subsequent assays. The knockdown sequences were synthesized and designed by GenePharma (Shanghai, China), and their detailed sequences are listed in Supplementary Table S1.

### 2.12. Colony Formation Assays

Cells in the logarithmic growth phase underwent treatment with trypsin, after which they were resuspended and plated into 6 cm dishes at a density of 1000 cells per dish. Following a culture period of 2 to 3 weeks, the colonies were fixed using a 4% paraformaldehyde solution, stained with crystal violet, and subsequently counted. The efficiency of colony formation was determined using the formula: (number of colonies/number of seeded cells) × 100%.

### 2.13. Cell Proliferation Assays

Cells in the logarithmic growth phase underwent trypsinization, after which they were counted and subsequently plated into 96-well plates at a density of 3000 cells per well (n = 6). Following incubation at specified time points (4, 24, 48, 72, 96, and 120 h), 10 µL of CCK-8 reagent was added to each well, and the plates were then incubated for an additional 2 h. Absorbance measurements were acquired at 450 nm using a microplate reader. The cell proliferation rate was calculated using the equation (A_d − A_blank_d)/(A_4 h − A_blank_4 h). All experiments were performed in triplicate to ensure the accuracy and reliability of the findings.

### 2.14. Transwell Assays

The study utilized Transwell chambers manufactured by Corning Inc., located in New York, NY, USA. Each chamber was filled with 150 microliters of a cell suspension containing 10,000 cells. In the lower compartment, 600 microliters of culture medium supplemented with 10% fetal bovine serum was introduced. After a 24 h incubation, non-migratory cells were eliminated, and the cells that had migrated were stained using a 0.25% crystal violet solution for a duration of 10 min. Subsequently, the chambers were rinsed with phosphate-buffered saline (PBS) and imaged. For the in vitro cell invasion assay, Transwell chambers that had been pre-coated with a matrix gel at a concentration of 100 μg/cm^2^, obtained from Thermo Fisher Scientific (Waltham, MA, USA), were employed.

### 2.15. Immunohistochemistry

Tissue sections (4–5 μm) from paraffin-embedded specimens were deparaffinized and subjected to heat-induced antigen retrieval in sodium citrate buffer (10 mM, pH 6.0). Sections were blocked with 5% normal serum in PBS and incubated overnight at 4 °C with primary antibodies. The following antibodies were used: anti-DDX39A (Abcam, 1:1000), anti-PBK (Abcam, 1:1000) and anti-CD161 (Abcam, 1:1000). After washing, sections were incubated with HRP-conjugated secondary antibodies (1:500) for 1 h at room temperature. Signals were developed using a DAB substrate kit, and nuclei were counterstained with hematoxylin. Images were acquired with a microscope. Quantification was performed on 3 random fields per sample from 100 biologically independent replicates using ImageJ software.

### 2.16. Evaluation of DDX39A and PBK Expression

The expression levels of DDX39A and PBK were independently assessed by two pathologists. blinded to clinical data. The staining intensity (0 = negative, 1 = weak, 2 = moderate, 3 = strong) and the percentage of positive tumor cells (0–100%) were recorded. A final immunoreactivity score (IRS) was calculated as [intensity × percentage], and samples were categorized into high-expression and low-expression groups using the median IRS as the cutoff. Based on this, 50 cases with high DDX39A, 50 with low DDX39A, 50 with high PBK, and 50 with low PBK were selected (total 200 unique samples; some cases with both high DDX39A and high PBK were allowed to maintain the total number).

### 2.17. Statistical Analysis

All statistical evaluations were conducted using R software alongside GraphPad Prism software version 10.5.0 (GraphPad Software, San Diego, CA, USA, www.graphpad.com). The assessment of differences between two groups was carried out using either Student’s *t*-test or the Wilcoxon rank-sum test, contingent upon the distribution of the data. For comparisons involving multiple groups, one-way ANOVA was applied, followed by Tukey post hoc analysis. The relationships between gene expression levels and the infiltration of immune cells were analyzed through Spearman correlation analysis. A two-tailed *p*-value of less than 0.05 was regarded as statistically significant.

## 3. Results

### 3.1. Identification of Toll-like Receptor-Related DEGs in ESCC Patients

**Figure 2:**
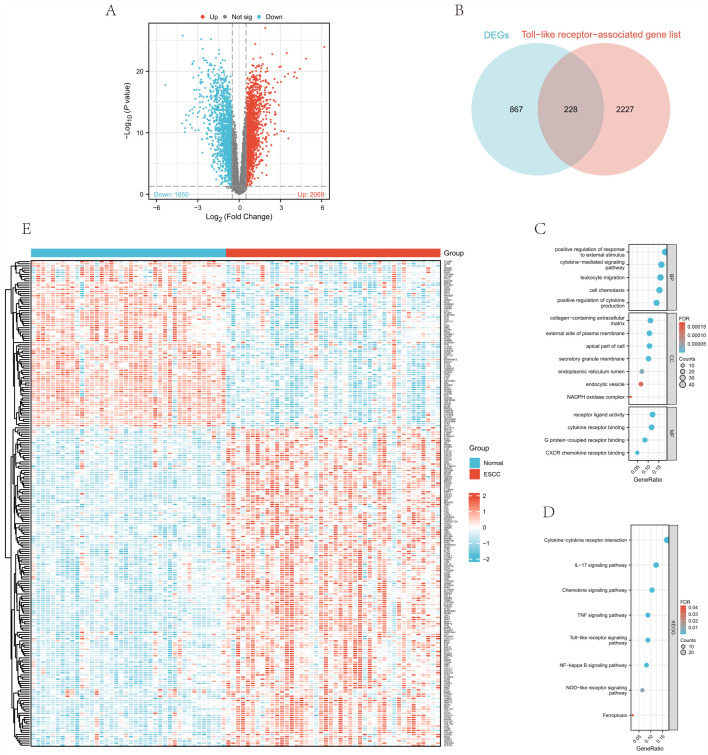
Identification of Toll-like receptor-associated DEGs for ESCC patients. (**A**) Distinct DEGs in the integrated dataset. (**B**) Identification of Toll-like receptor-associated DEGs. (**C**) Expression patterns of Toll-like receptor-associated DEGs. (**D**) GO enrichment of Toll-like receptor-associated DEGs. (**E**) KEGG enrichment of Toll-like receptor-associated DEGs.

In the integrated bulk profile of ESCC patients, we first identified 2069 up-regulated DEGs and 1650 down-regulated DEGs (Figure 2A). In addition, these DEGs were intersected with the Toll-like receptor gene list and identified 228 Toll-like receptor-associated DEGs (Figure 2B). We then estimated the expression patterns of these DEGs and corresponding molecular and biological functions (Figure 2C–E). As shown in Figure 2D,E, the pathways related to the interactions between cytokines and their receptors, cytokine-mediated signaling pathways, and lymphocyte migration were significantly enriched.

### 3.2. Identification of T Helper and Th17 Cell-Shared Module for ESCC Patients

**Figure 3:**
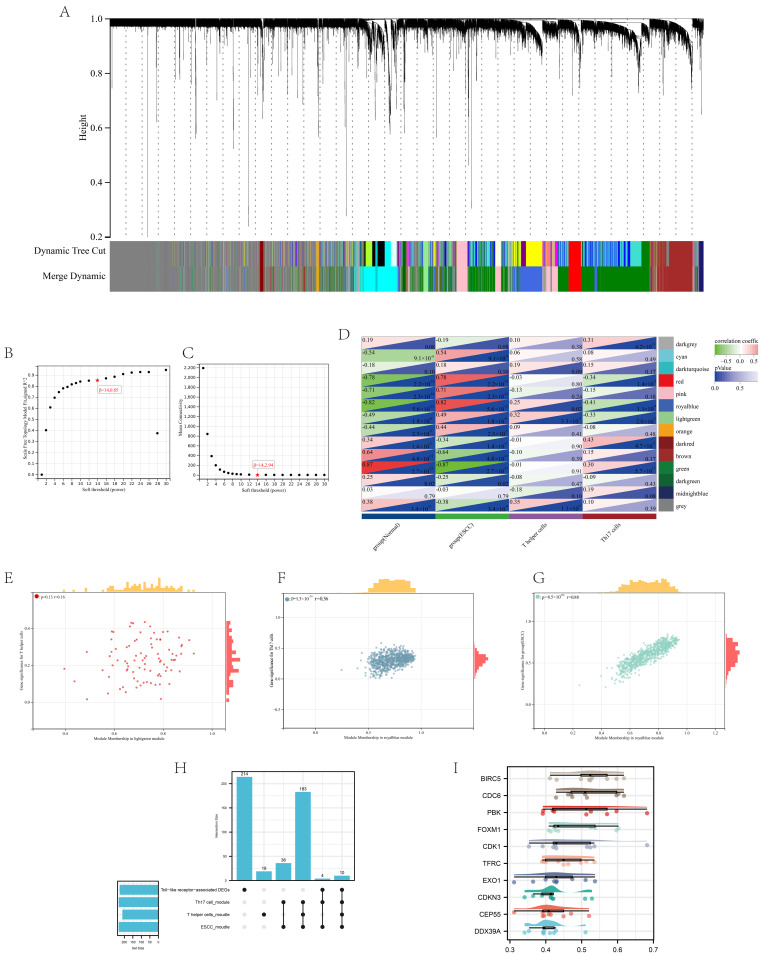
Identification of ThpT-associated DEGs for ESCC patients. (**A**) Gene co-expression modules with different colors under the gene tree. (**B**,**C**) Scatterplots of representative modules in the integrated dataset. (**D**) Module–trait relationship heatmap generated by WGCNA in the integrated dataset. (**E**–**G**) Correlation modules of WGCNA in the integrated dataset. (**H**) Intersection of ThpT-associated DEGs. (**I**) Friend analysis of ThpT-associated DEGs.

Using a combination of the ssGSEA immune infiltration algorithm and the integrated bulk profile, we constructed an unstructured co-expression network using WGCNA to identify modules most highly correlated with ESCC, as well as those correlated with Th17 and T helper cells. Based on the scale independence and average connectivity, we chose the soft threshold power β = 14, with scale independence of 0.85 and average connectivity of 2.94 (Figure 3B,C). The clustering dendrogram was generated for the ESCC and control groups (Figure 3A). By setting the module merging threshold to 0.25 and the minimum module size to 50, 14 gene co-expression modules represented by different colors were obtained, as shown in Figure 3D. We discovered that the royal blue, light green, and dark red modules were highly correlated with ESCC, T helper cells, and Th17 cells, respectively (Figure 3D–G). Indeed, we intersected Toll-like receptor signaling with the aforementioned highly correlated modules and discovered 10 ThpT-associated DEGs. The 10 genes mainly include BIRC5, CDC6, PBK, FOXM1, CDK1, TFRC1, EXO1, CDKN3, CEP55, and DDX39A (Figure 3H,I).

### 3.3. Identification of ThpT-Related Molecular Subgroups in ESCC Patients

**Figure 4:**
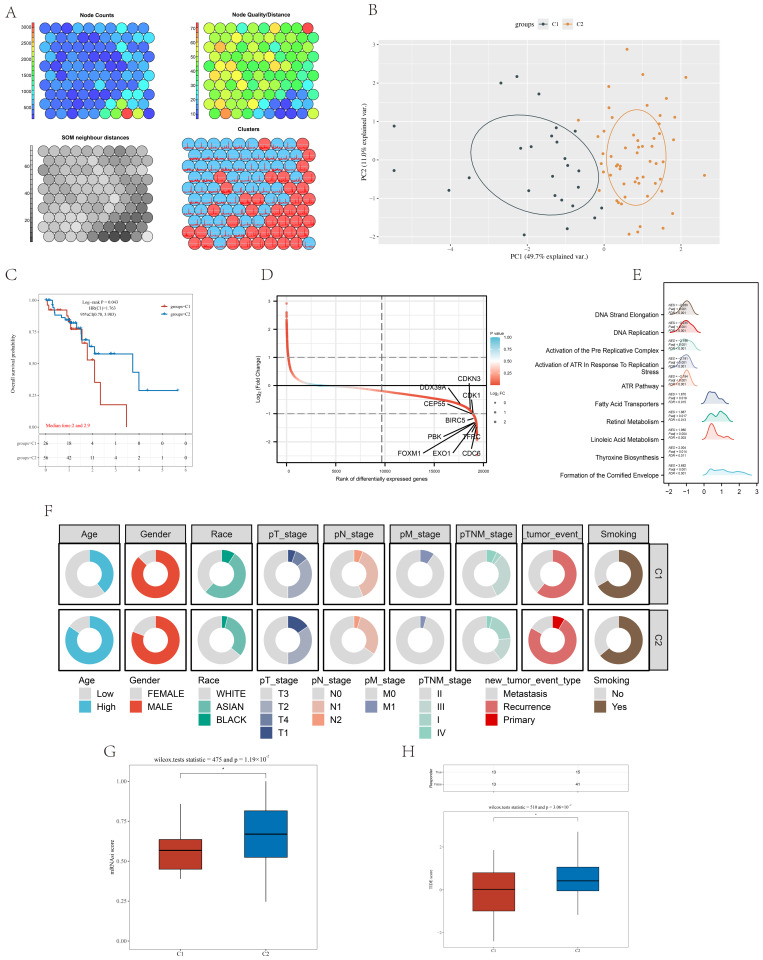
Identification of ThpT-associated subgroups for ESCC patients. (**A**) SOM deep learning clustering algorithm. (**B**) PCA plot of two subgroups. (**C**) KM analysis of two groups. (**D**) Clinical parameters of these two subgroups. (**E**) GSEA of these two groups. (**F**) Confirmation of clinical status among subgroups. (**G**) Tumor stemness analysis. (**H**) TIDE score of these two groups. * means *p* < 0.05.

To enhance the personalization of ESCC treatment, we applied the deep learning algorithm SOM in the TCGA-ESCC cohort to identify distinct ThpT-associated molecular subgroups based on the 10 ThpT-associated DEGs. We discovered 2 novel molecular groups, namely C1 and C2, and C2 illustrated the worst prognosis compared to C1 (Figure 4C). We next identified the differences in ThpT-associated DEGs, clinical parameters, and molecular functions between these two groups. The results show that the tumor recurrence rate in group C2 is higher than that in group C1 (Figure 4D–F). Next, we examined tumor stemness and TIDE score between these two groups. The results revealed that both the tumor stemness index and the TIDE score were significantly higher in the C2 group compared to the C1 group (Figure 4G,H).

### 3.4. LASSO-Cox Regression for the ThpT-Related Prognostic Model Construction in ESCC Patients

**Figure 5:**
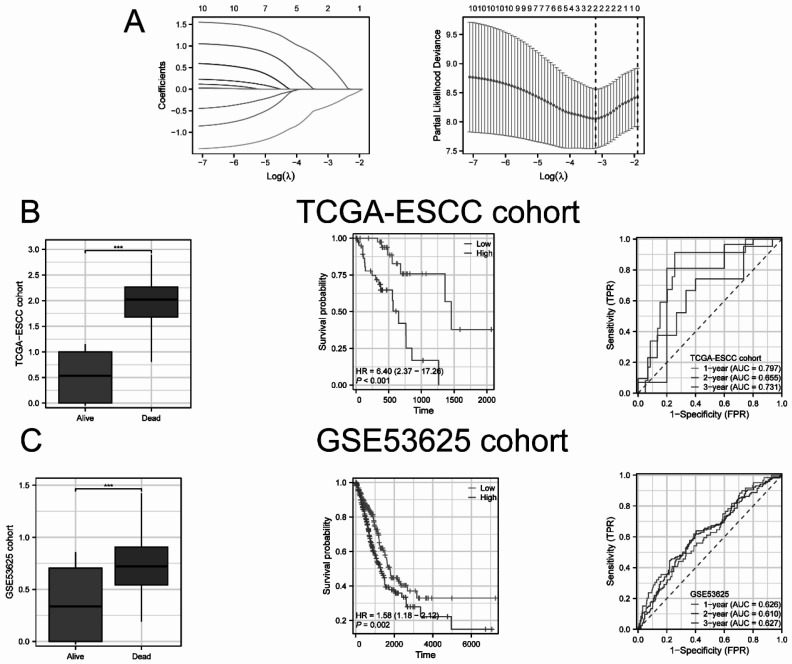
Identification of ThpT-associated molecular subgroups for ESCC patients. (**A**) Variation characteristics of Lasso results. (**B**) ThpT-associated prognostic model construction in the TCGA-ESCC cohort. (**C**) ThpT-associated prognostic model construction in the GSE53625 cohort. *** means *p* < 0.001.

We performed LASSO-Cox regression analysis on 10 ThpT-related DEGs and constructed a LASSO regression model based on the TCGA-ESCC cohort as the training set and the GSE53625 cohort as the validation set (Figure 5A). Next, we cross-evaluated the prognostic model efficacy and accuracy in the TCGA-ESCC and GSE53625 cohorts (Figure 5B,C). Indeed, in both the TCGA-ESCC and GSE53625 cohorts, the high-risk model indicated an unsatisfactory prognostic outcome. Furthermore, after Lasso-Cox regression analysis, we [identified] that DDX39A and PBK can be considered ThpT-related hub genes.

### 3.5. Analysis of ThpT-Hub Molecular and Immune Signatures

**Figure 6:**
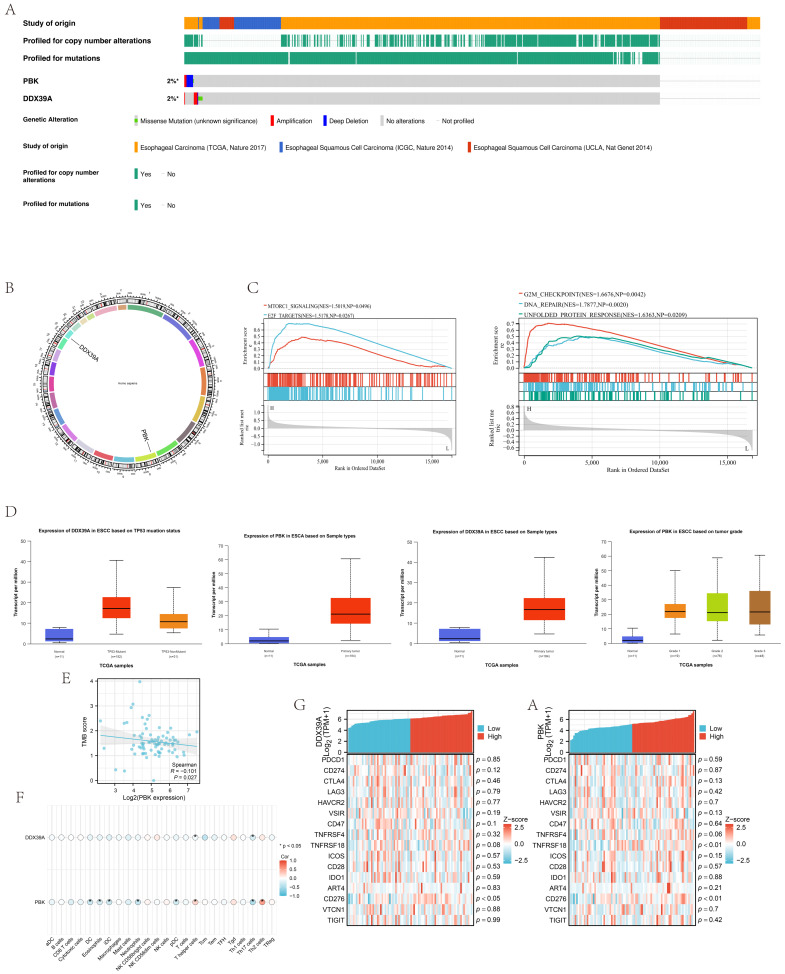
Association of PBK and DDX39A with immune and molecular features. (**A**) Genetic variation analysis of PBK and DDX39A in ESCC patients. (**B**) Chromatin localization of PBK and DDX39A in ESCC patients. (**C**) Single-gene GSEA enrichment analysis of PBK and DDX39A in ESCC patients. (**D**) ESCC clinical parameters with PBK and DDX39A expression in ESCC patients. (**E**) The relationship of PBK with MSI in ESCC patients. (**F**) The association of immune cells with PBK and DDX39A in ESCC patients. (**G**) The association of immune checkpoints with PBK and DDX39A in ESCC patients. * means *p* < 0.05.

First, based on genetic variation analysis, we discovered that PBK and DDX39A have no significant mutations in ESCC (Figure 6A). Next, we also evaluated PBK and DDX39A chromatin localization to gain insights into their genetic roles (Figure 6B). Furthermore, we evaluated the molecular and biological functions of PBK and DDX39A in ESCC, as shown in Figure 6C. DDX39A is associated with the G2M checkpoint, DNA repair, and the unfolded protein response pathways; PBK is related to mTORC1 signaling and E2F target pathways. Additionally, the expression of PBK and DDX39A was assessed according to clinical parameters of ESCC patients. The higher the expression of DDX39A is, the higher the probability of TP53 mutation in esophageal squamous cell carcinoma also is. Compared with normal tissues, DDX39A and PBK are upregulated in esophageal squamous cell carcinoma (Figure 6D). Importantly, we also discovered a negative correlation between PBK and the MSI score (Figure 6E). The association between PBK and DDX39A with immune cell proportion and checkpoint expressions in ESCC was also assessed. The results show that the expression of DDX39A is negatively correlated with the infiltration of T helper cells and Th17 cells; the expression of PBK is negatively correlated with the infiltration of DCs, eosinophils, neutrophils, pDCs, and Th17 cells, but positively correlated with the infiltration of T helper cells and Th2 cells (Figure 6F). The expression of DDX39A is positively correlated with the expression of immune checkpoint CD276; the expression of PBK is also positively correlated with the expressions of immune checkpoints CD276 and TNFRSF18 (Figure 6G).

### 3.6. Landscape of ThpT-Hub Signature at the Single-Cell Level in ESCC

**Figure 7:**
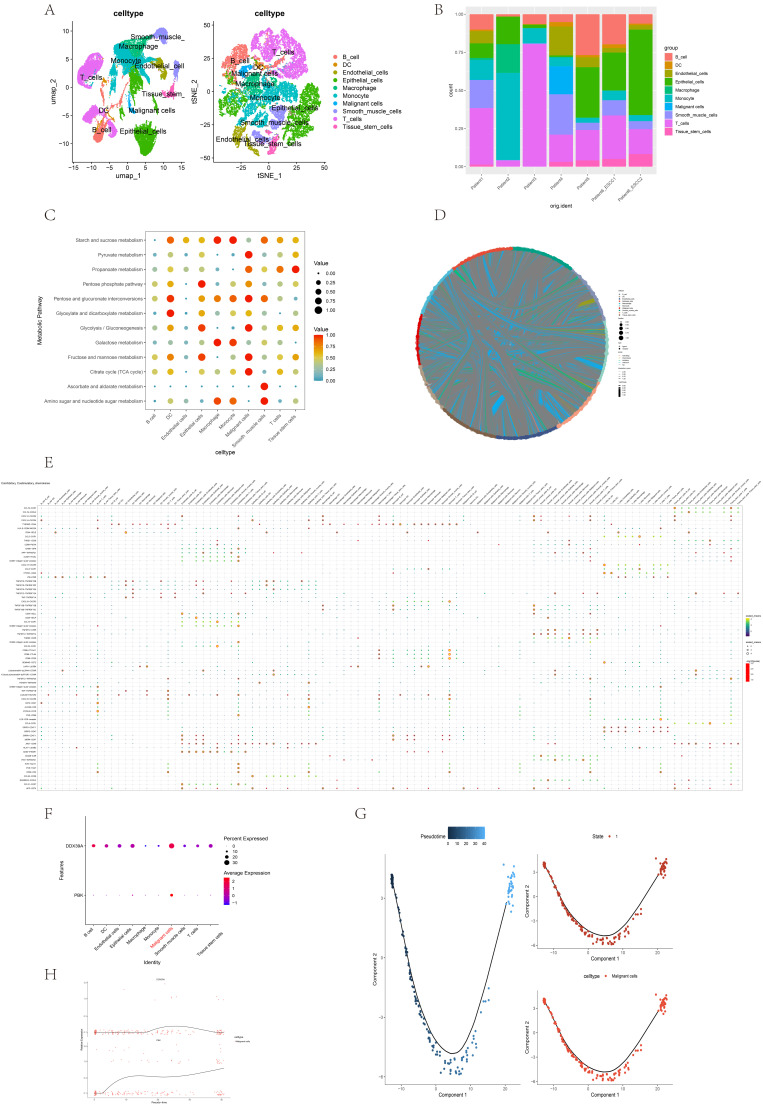
Landscape of ESCC single cell data and the role of PBK and DDX39A at the single-cell level in ESCC. (**A**,**B**) Single-cell-level annotation of ESCC samples obtained via UMAP and t-SNE analysis. (**B**) Expression of PBK and DDX39A in cells from different cell types. (**C**) Metabolic pathways in the 10 cell types. (**D**,**E**) Cell communication among the 10 cell types, derived from the annotation results. (**F**) Expression of PBK and DDX39A in cells from different cell types. (**G**,**H**) Pseudo-time plot showing the expression changes in PBK and DDX39A in different differentiation stages of malignant cells.

scRNA-seq data used in this study were retrieved from the GSE188900 dataset. First, the data were subjected to normalization and quality control (QC) (Figure S1A–C). To better characterize the cell types, we classified the cells into 32 distinct cell clusters using the expression of known markers (Figure S1D,E). Next, after being annotated, 10 distinct cell types were confirmed, including B cells, DC cells, endothelial cells, epithelial cells, macrophages, monocytes, malignant cells, smooth muscle cells, T cells and tissue stem cells (Figure 7A,B). We analyzed PBK and DDX39A expression across different cell types and found that PBK and DDX39A were highly expressed in malignant cells (Figure 7F). Cell–cell communication analysis of the 10 cell types revealed that malignant cells exhibited robust interactions with the other 9 cell types, suggesting a critical role of malignant cells in intercellular communication (Figure 7D). Indeed, the cell–cell communication pathways among these 10 cell types were also enriched (Figure 7E). Additionally, we analyzed the metabolic microenvironment of the 10 cell types (Figure 7C). Finally, we investigated the dynamic changes in PBK and DDX39A expressions along the differentiation trajectory of malignant cells. The results show that the expression levels of PBK and DDX39A are high at the early stage of malignant cell differentiation (Figure 7G,H). Taken together, these findings provide single-cell-level evidence that PBK and DDX39A may act as a key node in the functional regulatory network of malignant cells.

### 3.7. Mechanisms of the ThpT-Hub Signature in Targeted Cells from ESCC Single-Cell Transcriptomic Data

**Figure 8:**
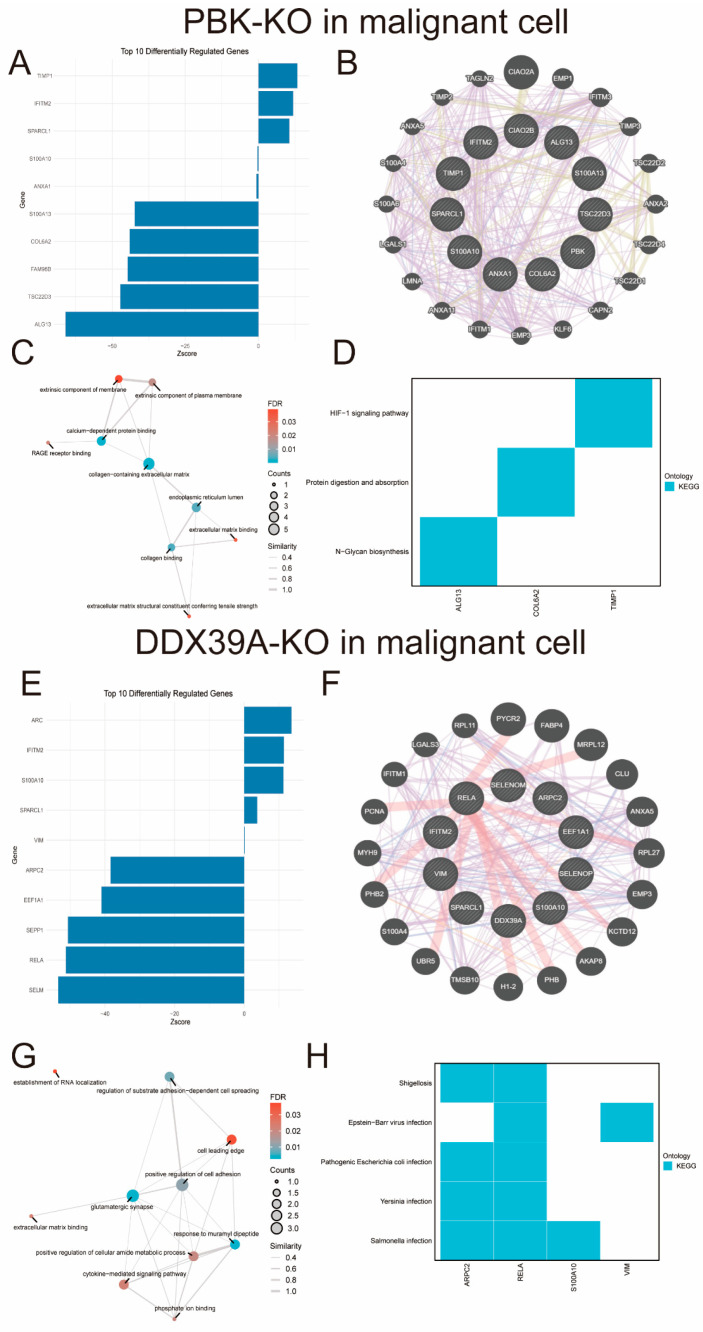
Regulatory role of PBK and DDX39A at the single-cell level in malignant cells of ESCC patients. (**A**) Top 10 DEGs after PBK KO in malignant cells. (**B**) GeneMANIA results of the top 10 DEGs after PBK KO in malignant cells. (**C**,**D**) GO and KEGG analysis of PBK KO in malignant cells. (**E**) Top 10 DEGs after DDX39A KO in malignant cells. (**F**) GeneMANIA results of the top 10 DEGs after DDX39A KO in malignant cells. (**G**,**H**) GO and KEGG analysis of DDX39A KO in malignant cells.

To gain insights into the functional role of PBK and DDX39A at the single-cell level, we performed single-cell targeted gene knockout (KO). We illustrated the top 10 DEGs after KO of PBK and DDX39A in malignant cells of the ESCC tumor microenvironment (Figure 8A,E). In addition, we also investigated the regulatory interactions and corresponding molecular and biological functions to gain a deeper understanding of targeted genes in malignant cells after PBK and DDX39A KO. Based on KEGG functional analysis, the results showed that the differentially expressed genes ALG13, COL6A2, and TIMP1 after PBK knockout were respectively enriched in N-glycan biosynthesis, protein digestion and absorption, and the HIF-1 signaling pathway (Figure 8B–D). KEGG enrichment analysis revealed that following DDX39A knockout, the differentially expressed genes—ARPC2, RELA, S100A10, and VIM—were significantly enriched in infection-related pathways, including shigellosis and Epstein–Barr virus infection (Figure 8F–H).

### 3.8. The Association of ThpT-Hub Signature with ESCC Cancer Progression and Tumor Microenvironment

**Figure 9:**
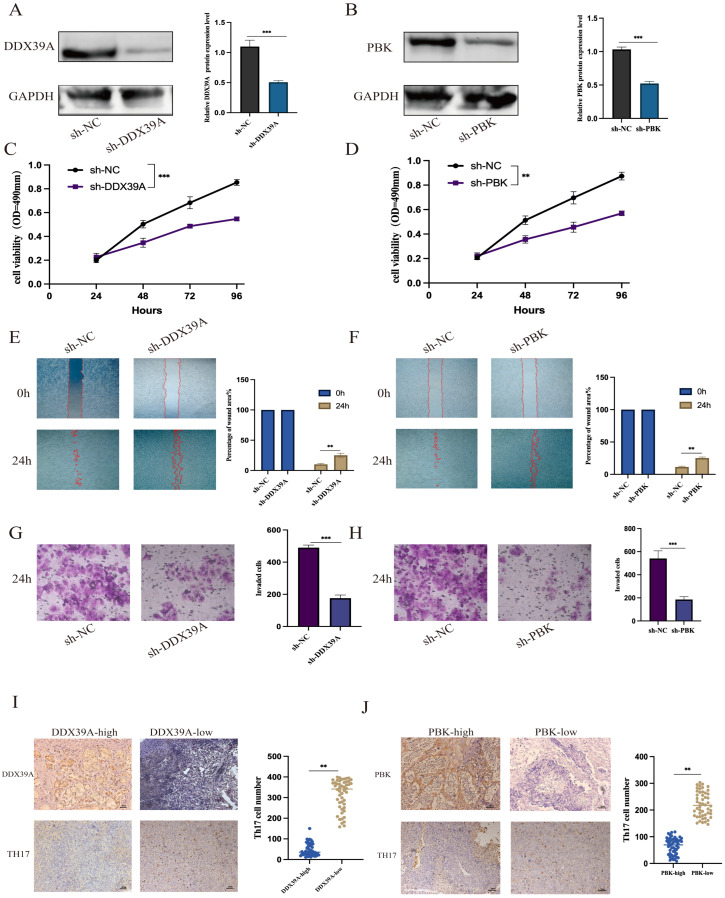
The association of ThpT-hub signature with ESCC cancer progression and tumor microenvironment. (**A**,**B**) The protein levels in KYSE150 cell lines after DDX39A and PBK knockdown. (**C**,**D**) CCK-8 analysis of KYSE150 cell viability under DDX39A and PBK silencing. (**E**,**F**) Wound healing assay results of knocking down DDX39A and PBK in the KYSE150 cell line. (**G**,**H**) Transwell analysis of KYSE150 cell migration under DDX39A and PBK silencing. (**I**,**J**) Using immunohistochemistry, the expression level of DDX39A in esophageal cancer tissues and the infiltration level of Th17 cells were respectively detected. *** means *p* < 0.001; ** means *p* < 0.01.

To further investigate the role of the Th17-hub signature DDX39A and PBK in ESCC, we separately constructed DDX39A and PBK knockdown stable cell lines derived from KYSE150 cells (Figure 9A,B). To assess the effect of DDX39A and PBK on cancer cell proliferation, the CCK-8 assay was used (Figure 9C,D). DDX39A and PBK knockdown inhibited cell growth. The wound-healing assay showed that cells with knockdown of DDX39A and PBK exhibited significantly slower wound closure (Figure 9E,F). Additionally, Transwell assays revealed that knockdown of DDX39A and PBK markedly decreased the invasion capabilities of ESCC cells (Figure 9G,H). These results indicated that DDX39A and PBK promote the progression of ESCC by enhancing cell proliferation, migration, and invasion. Furthermore, from 200 paraffin-embedded samples of esophageal squamous cell carcinoma, we selected 50 cases each with high expression of DDX39A and PBK, as well as 50 cases each with low expression of DDX39A and PBK. Using immunohistochemical staining (Figure 9I,J), we detected the infiltration number of Th17 cells within the tumors. The results showed that high expression of DDX39A and PBK in esophageal squamous cell carcinoma tissues was associated with reduced infiltration of Th17 cells.

### 3.9. Targeted Drug Enrichment and Molecular Docking for the Treatment of ESCC

**Figure 10:**
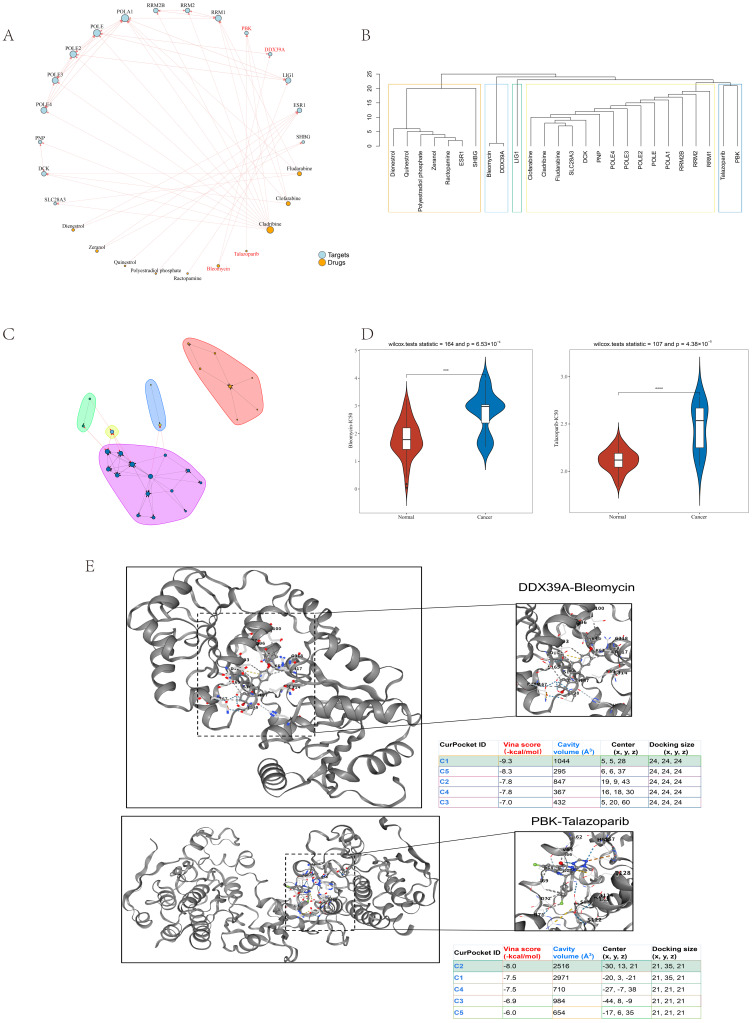
Drug reproposing framework for ESCC patients. (**A**–**C**) Drug–gene network construction. (**D**) Drug sensitivity analysis. (**E**) Molecular docking validation. *** means *p* < 0.001; **** means *p* < 0.0001.

After construction of the drug–gene interaction network, we discovered that bleomycin and talazoparib were two optimal drug repurposing strategies for treatment of ESCC by targeting DDX39A and PBK (Figure 10A–C). Next, ridge regression was performed to identify the high sensitivity of these two drugs for ESCC treatment (Figure 10D). In addition, these two drugs have satisfactory binding affinity with the C1 cavity pocket of DDX39A (−9.3 kcal/mol) and the C2 cavity pocket of PBK (−8.0 kcal/mol) (Figure 10E).

## 4. Discussion

ESCC remains a significant global health challenge, with limited therapeutic options and poor prognosis for advanced stages [24]. Current treatment strategies often fail to account for the complex tumor microenvironment and the immune landscape, which underscores the necessity for innovative approaches that can enhance patient outcomes [52,53,54,55,56].

CD4+ T cells are pivotal activators of both innate and adaptive immunity, playing a critical role in host defense against pathogens while also mediating pathological processes in allergic disorders, autoimmunity, and tumorigenesis [57]. Th17 cells represent a distinct CD4+ T helper lineage separate from classical Th1 and Th2 subsets. Initially identified by Harrington in mice as IL-17-producing CD4+ T cells, this population was subsequently designated Th17 cells [58]. Their differentiation is coordinately regulated by the lineage-defining transcription factors RORγt and STAT3 [28,29] and is highly dependent on the cytokine milieu. IL-6 and TGF-β act synergistically to drive Th17 polarization [30]. The concentration gradient of TGF-β dictates lineage commitment: lower concentrations promote RORγt expression and Th17 differentiation, whereas higher concentrations suppress RORγt, induce Foxp3, and divert development towards regulatory T (Treg) cells [31,32]. In the presence of TGF-β, IL-21 can substitute for IL-6 to induce Th17 differentiation [32]. Functionally, Th17 cells exert pro-inflammatory effects primarily through secretion of their signature cytokine, IL-17 [33,34]. The Th17/IL-17 axis is established in the pathogenesis of autoimmune diseases such as asthma, systemic lupus erythematosus, and rheumatoid arthritis, although its role in tumor biology remains contentious and an active area of investigation [35,36].

First, we obtained Toll-like receptor-related differentially expressed genes in esophageal squamous cell carcinoma through differential analysis. Then, we identified the T helper and Th17 cells shared module genes in esophageal squamous cell carcinoma by using WGCNA. Further, to identify ThpT-related molecular subtypes in esophageal cancer (ESCC), we adopted the self-organizing map (SOM) deep learning algorithm based on 10 ThpT-related differentially expressed genes in the TCGA cohort. Meanwhile, we conducted LASSO-Cox regression analysis on the 10 ThpT-related differentially expressed genes and constructed a LASSO regression model based on the TCGA-ESCC cohort and the GSE53625 cohort as the training set. Finally, we screened out two significantly differentially expressed genes, DDX39A and PBK.

DDX39A, a member of the DEAD-box RNA helicase family, has been demonstrated to interact with ALY, CIP29, and FUS/TLS, thereby contributing to the regulation of transcription, splicing, and RNA export [37]. DDX39A is overexpressed and promotes cellular proliferation and metastasis in hepatocellular carcinoma, breast cancer, and clear cell renal cell carcinoma [38,39,40]. Elevated DDX39 expression has been associated with chemoresistance to doxorubicin and gemcitabine in breast cancer and pancreatic cells [38,41,59]. In clear cell renal cell carcinoma, DDX39 expression positively correlates with several immune inhibitory markers and may predict an unfavorable response to immune checkpoint therapy [39]. However, the expression profile, functional roles, and regulatory mechanisms of DDX39A in esophageal squamous cell carcinoma remain elusive. We evaluated the proportion of immune cells and the expression of immune checkpoints in esophageal squamous cell carcinoma (ESCC) in relation to DDX39A expression. We found that the expression of DDX39A was inversely correlated with the tumor infiltration of T helper cells and Th17 cells and positively correlated with the expression of the immune checkpoint CD276. Additionally, through single-cell analysis, we discovered that DDX39A was mainly highly expressed in tumor epithelial cells. To analyze the biological functions of PBK and DDX39A at the single-cell level, we conducted single-cell targeted gene knockout experiments, which revealed that DDX39A knockout could increase the expression of ARC, IFITM2, S100A10, and SPARCL1, while reducing the expression of ARPC2, EEF1A1, SEPP1, and RELA. In vitro experiments showed that inhibiting DDX39A expression could significantly reduce the proliferation, migration, and invasion abilities of esophageal squamous cell carcinoma cells. Moreover, using immunohistochemistry on clinical patient paraffin samples, we found that DDX39A expression was negatively correlated with the infiltration of Th17 cells in esophageal squamous cell carcinoma.

PBK, a serine/threonine kinase, regulates the G2/M checkpoint, mitotic spindle integrity, and chromosomal stability [42]. Its overexpression has been documented in multiple malignancies, including endometrial hyperplasia and low-grade endometrial carcinoma, where PBK expression levels can discriminate indolent from aggressive lesions [43]. Recent multi-omics profiling further demonstrates that PBK operates through the p53 and FOXO signaling pathways, modulates cytoskeletal dynamics, and correlates with immune infiltration in breast cancer, underscoring its pleiotropic oncogenic potential [44,60]. We evaluated the role of PBK in esophageal squamous cell carcinoma (ESCC) in relation to immune cell proportions and checkpoint expression. PBK expression showed a positive correlation with intratumoral infiltration of helper T cells and Th2 cells but was negatively correlated with infiltration of Th17 cells and dendritic cells. Conversely, PBK expression was positively associated with the expression of immune checkpoints TNFRSF18 and CD276. Single-cell analysis revealed that PBK is predominantly highly expressed in tumor epithelial cells. To investigate its functional role at single-cell resolution, we performed targeted knockout of PBK, which resulted in upregulation of TIMP1, IFITM2, and SPARCL1, along with downregulation of S100A13, COL6A2, and FAM96B. In vitro assays further demonstrated that PBK suppression significantly impaired the proliferation, migration, and invasion capacities of ESCC cells. Immunohistochemistry on clinical specimens confirmed an inverse correlation between PBK expression and Th17 cell infiltration in tumor tissues. Finally, by constructing a drug–gene interaction network, we identified talazoparib as a potential therapeutic agent targeting PBK in ESCC.

Finally, by constructing a drug–gene interaction network, we confirmed that bleomycin and talazoparib are among the best drugs for targeting DDX39A and PBK in the treatment of ESCC.

Bleomycin (BLM), as a type of antibiotic, can bind to the DNA of tumor cells, interfering with DNA replication and transcription, thereby inhibiting the proliferation and growth of tumor cells and achieving tumor suppression. Clinically, it is mainly used in combination with anticancer drugs such as doxorubicin and has good therapeutic effects on head and neck squamous cell carcinoma, nasopharyngeal carcinoma, malignant lymphoma, esophageal cancer, colon cancer, vulvar cancer, testicular cancer, lung cancer, Hodgkin’s disease, etc. [61,62]. In the treatment of breast cancer, the use of chemotherapeutic drugs delivered through electrical pulses, such as injecting bleomycin into the tumor or intravenously, has shown better efficacy and prognosis for elderly patients with metastatic breast cancer, and it is positively correlated with their physical and mental condition [63]. However, bleomycin is not a substance that can freely pass through the cell membrane; instead, it is absorbed by cells through specific receptors. Nevertheless, the expression levels of the relevant receptors for direct absorption of bleomycin in esophageal cancer tumor cells are still unclear at present. Therefore, based on the absorption pathway of bleomycin in esophageal tumor cells, its clinical translational significance still needs to be further explored.

Talazoparib is a potent PARP inhibitor that inhibits the activity of PARP enzymes and traps PARP at DNA damage sites, interfering with the repair of DNA single-strand breaks. Eventually, it triggers synthetic lethal effects in tumor cells with homologous recombination repair deficiencies (such as BRCA1/2 mutations) [64,65,66]. In terms of clinical application, based on the phase III EMBRACA trial, it has been approved for the treatment of HER2-negative locally advanced or metastatic breast cancer with germline BRCA mutations [67]. Additionally, based on the results of the TALAPRO-2 trial, the combination of talazoparib and enzalutamide has been proven to significantly improve radiographic progression-free survival and overall survival of patients [68,69,70]. Currently, its application in prostate cancer has expanded to patients regardless of the status of homologous recombination repair gene mutations, but those with BRCA mutations benefit more significantly [71,72,73]. However, the therapeutic effect of talazoparib in esophageal squamous cell carcinoma has not been clearly defined. In this study, by constructing a drug–gene interaction network, it was found that PBK is a potential target for talazoparib in the treatment of esophageal squamous cell carcinoma, providing a theoretical basis for the use of talazoparib in the treatment of esophageal squamous cell carcinoma.

## 5. Conclusions

In summary, our study provides a novel clinical translation strategy for personalized and precision medicine in ESCC patients, highlighting the therapeutic potential of bleomycin and talazoparib. The identification of a Thp-associated predictive model serves as a guiding factor for forecasting patient clinical outcomes and treatment decisions, opening avenues for future research. To fully realize the implications of our findings, pre-clinical studies and clinical trials are essential to confirm the prognostic model and the role of the identified mechanisms of hub genes in ESCC progression. This will ultimately enhance the clinical applicability of our results and contribute to improved patient outcomes.

## Data Availability

4 bulk cohorts of ESCC, namely GSE161533, GSE17351, GSE77861, and GSE100942, were sourced from the Gene Expression Omnibus (GEO) database.

## Supplementary Materials

The following supporting information can be downloaded at: https://www.mdpi.com/article/10.3390/bioengineering13060622/s1, Table S1. The detailed sequences of shRNA knockdown fragments. Figure S1. Normalization and quality control of scRNA-seq.
