# Supplementary material for: Artificial Intelligence-Driven Construction of Predictive and Druggable Frameworks to an In Silico Bioengineering Evidence Support for Therapy of Esophageal Squamous Cell Carcinoma Patients: Insights from a Toll-like Receptor Signal with Th17 and T Helper Microenvironment
Source: Bioengineering (Basel). 2026 May 26;13(6):622. doi: 10.3390/bioengineering13060622 (PMC13296311; doi:10.3390/bioengineering13060622)
Supplement: Supplementary file 1 [file bioengineering-13-00622-s001.zip › bioengineering-4190279-supplementary.pdf]

bioengineering

MDPI

Article

Artificial Intelligence-Driven Construction of Predictive and Druggable Frameworks to an In Silico Bioengineering Evidence Support for Therapy of Esophageal Squamous Cell Carcinoma Patients: Insights from a Toll-like Receptor Signal with Th17 and T Helper Microenvironment

Bo Liu [1,2,†], Jiazhou Xiao [1,2,†], Xuan Tao [3,4,†] and Xu Li [1,2,*]

1 Department of Thoracic Surgery, The First Affiliated Hospital, Fujian Medical University, Fuzhou 350004, China
2 Department of Thoracic Surgery, National Regional Medical Center, Binhai Campus of the First Affiliated Hospital, Fujian Medical University, Fuzhou 350212, China
3 Department of Pathology, The First Affiliated Hospital, Fujian Medical University, Fuzhou 350212, China
4 Department of Pathology, National Regional Medical Center, Binhai Campus of the First Affiliated Hospital, Fujian Medical University, Fuzhou 350212, China
* Correspondence: lixu1967@hotmail.com; Tel.: +86-(0591)-87982102
† These authors contributed equally to this work.

Supplementary Materials

Supplementary Table S1. The detailed sequences of shRNA knockdown fragments

Group	Target gene	ShRNA sequence (5′→3′)
sh-NC	Negative control	CTCTTCTCCATAGCACTAAT
sh-DDX39A	DDX39A	GCGAGTCAACATCGTCTTTAA
sh-PBK	PBK	**CTCTTCTCTGTATGCACTAAT**

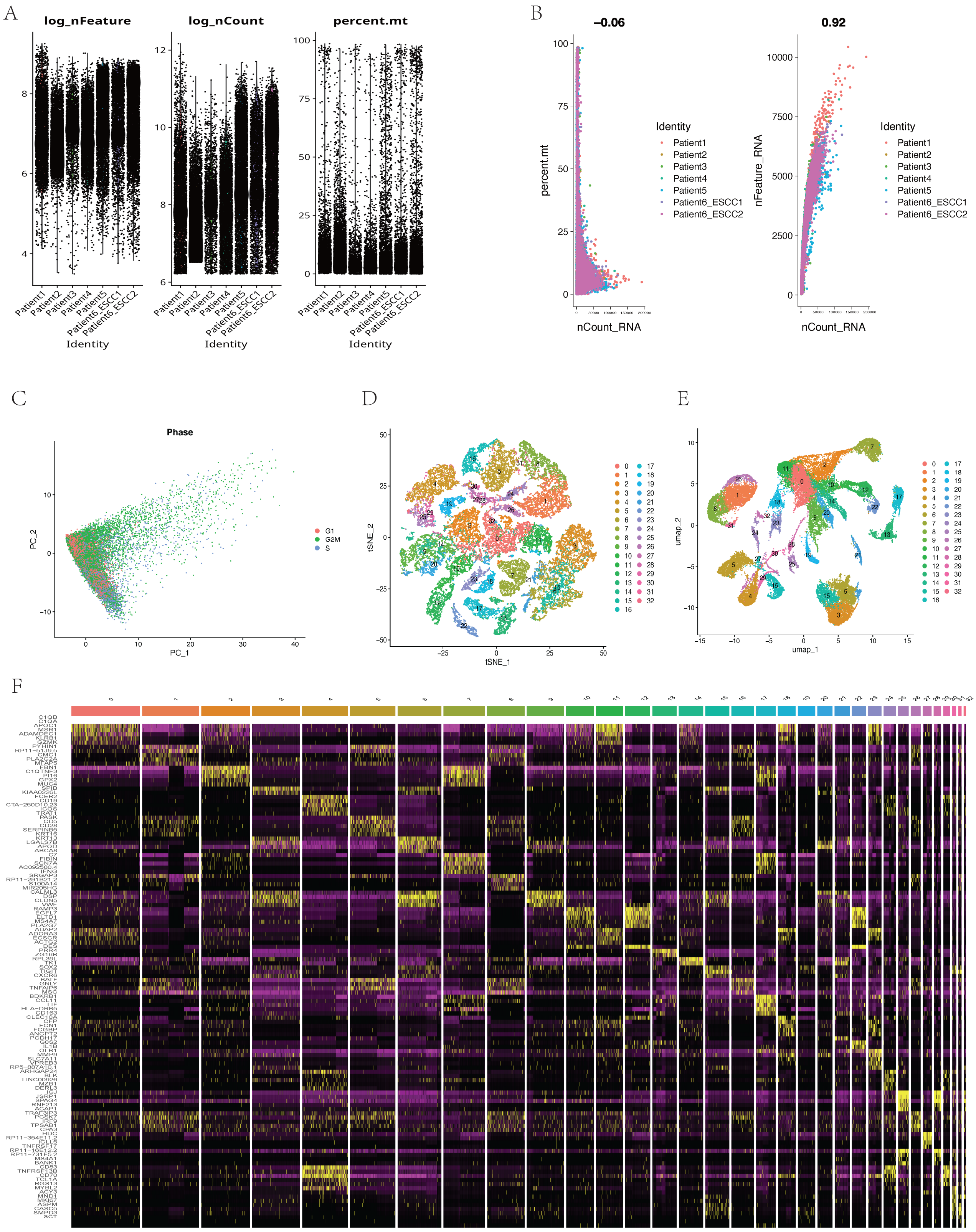

Supplementary Figure S1. Normalization and quality control of scRNA-seq. (A) Single-cell sequencing depth, counts and fraction of reads mapped to mitochondrial genes in GSE188900. **(B)** Correlation of gene count and percent of mitochondrial genes and features. **(C)** Scatter plot of the distribution of clusters. **(D-E)** Dimensionality reduction using t-SNE and UMAP. **(F)** Stacked bar chart showing the relative abundance of each cell type.

Disclaimer/Publisher's Note: The statements, opinions and data contained in all publications are solely those of the individual author(s) and contributor(s) and not of MDPI and/or the editor(s). MDPI and/or the editor(s) disclaim responsibility for any injury to people or property resulting from any ideas, methods, instructions or products referred to in the content.
