# Supplementary material for: Artificial Intelligence-Driven Construction of Predictive and Druggable Frameworks to an In Silico Bioengineering Evidence Support for Therapy of Esophageal Squamous Cell Carcinoma Patients: Insights from a Toll-like Receptor Signal with Th17 and T Helper Microenvironment
Source: Bioengineering (Basel). 2026 May 26;13(6):622. doi: 10.3390/bioengineering13060622 (PMC13296311; doi:10.3390/bioengineering13060622)
Supplement: Supplementary file 1 [file figure S-1.pdf › figure S-1.pdf]

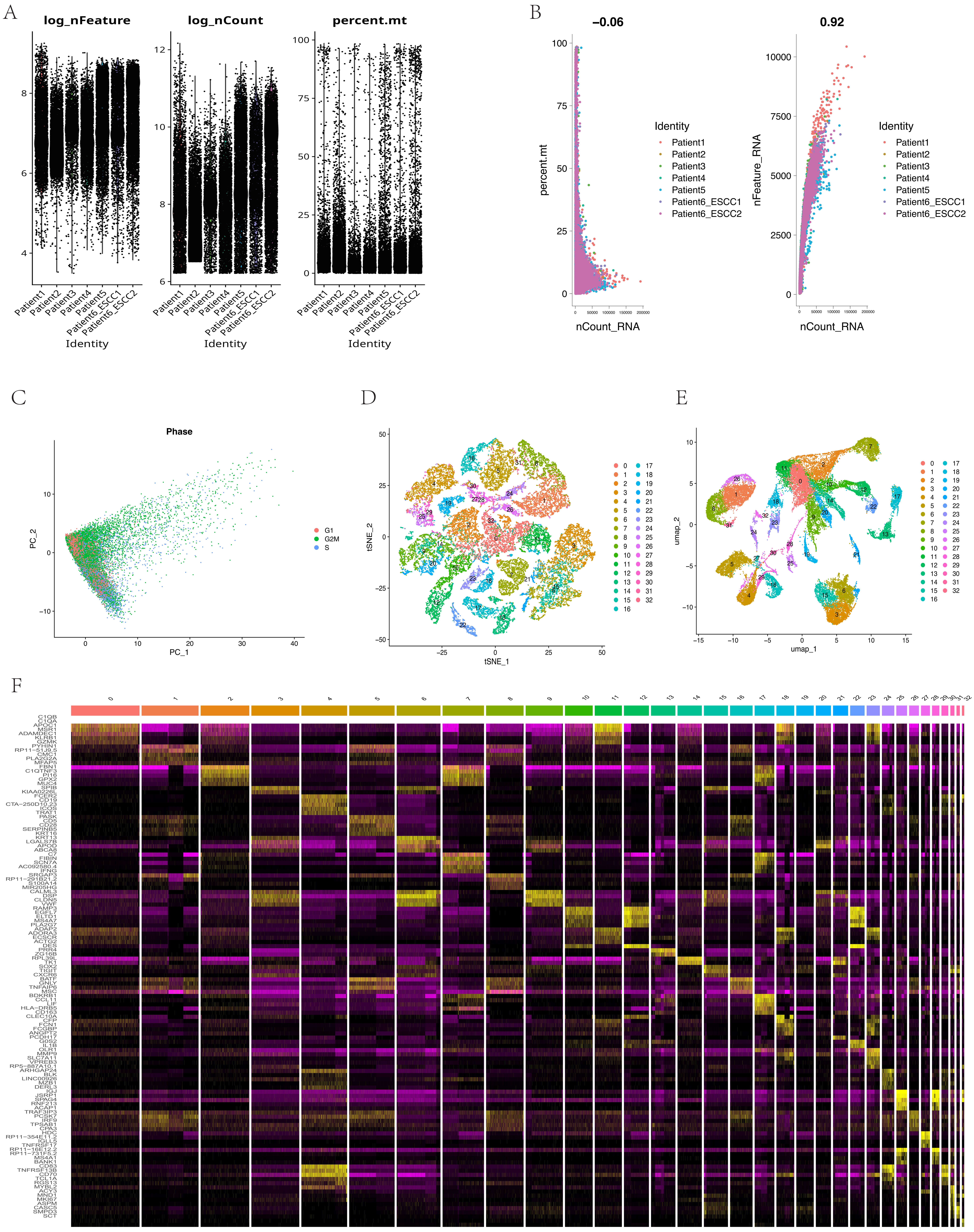
A
log_nFeature
log_nCount
percent.mt
Identity
Patient1
Patient2
Patient3
Patient4
Patient5
Patient6_ESCC1
Patient6_ESCC2
B
-0.06
0.92
percent.mt
nFeature_RNA
nCount_RNA
Identity
C
Phase
PC_1
PC_2
G1
G2M
S
D
tSNE_1
tSNE_2
E
umap_1
umap_2
F
